# Copper deposition in Wilson’s disease causes male fertility decline by impairing reproductive hormone release through inducing apoptosis and inhibiting ERK signal in hypothalamic-pituitary of mice

**DOI:** 10.3389/fendo.2022.961748

**Published:** 2022-08-05

**Authors:** Tingting Wang, Limin Wu, Qiuying Chen, Kuiyu Chen, Fang Tan, Jiabo Liu, Xiang Liu, Hui Han

**Affiliations:** ^1^ Encephalopathy Center, The First Affiliated Hospital, Anhui University of Traditional Chinese Medicine, Hefei, China; ^2^ Reproductive and Genetic Branch, The First Affiliated Hospital of University of Science and Technology of China (USTC), Division of Life Sciences and Medicine, University of Science and Technology of China, Hefei, China

**Keywords:** Wilson’s disease, reproductive neuroendocrine, infertility, hypothalamic–pituitary–testicular axis, apoptosis, extracellular regulatory protein kinase1/2 (ERK1/2)

## Abstract

Wilson’s disease (WD) is an autosomal recessive disorder of copper metabolism characterized by liver and central nervous system dysfunction. Considerable evidence suggests that infertility is also very common in male patients with WD, but the exact molecular mechanisms involved remain unknown. In order to further investigate the pathological changes in the hypothalamic-pituitary-testicular (HPT) axis and its mechanisms, mice were divided into the normal control group (NC), WD model TX mice group (WD), dimercaptosuccinic acid–treated TX mice group (DMSA), and pregnant horse serum gonadotropin–treated TX mice group (PMSG). The copper content and morphology of hypothalamus and pituitary tissues, the ultrastructure and apoptosis of hypothalamus neurons and pituitary gonadotropin cells, the serum levels of reproductive hormones, and the pregnancy rate and litter size of the female mice were studied. The expression of apoptosis-related proteins and the phosphorylation of extracellular regulatory protein kinase (ERK) 1/2 in the hypothalamus and pituitary were detected. The results showed that the copper content was significantly increased in the WD group, and the histopathological morphology and ultrastructure of the hypothalamus and pituitary were damaged. The levels of the gonadotropin-releasing hormone, the follicle-stimulating hormone, the luteinizing hormone, and testosterone were significantly decreased. The apoptosis rate in the hypothalamus and pituitary was significantly increased. The expressions of proapoptotic proteins Bax and Caspase-3 were significantly increased, the expression of the anti-apoptotic protein Bcl-2 was significantly decreased, and the phosphorylation level of ERK1/2 was significantly decreased. Fertility is significantly reduced. After DMSA intervention, the hypothalamus tissue copper content decreased, the hypothalamus and pituitary tissue morphology and ultrastructure were improved, cell apoptosis was alleviated, the expression of Bax and Caspase-3 was significantly decreased, the expression of Bcl-2 was significantly increased, and the reproductive hormone level, phosphorylation level, and fertility were increased. Fertility was preserved after treatment with PMSG in male TX mice. These results suggest that copper deposition in WD causes male fertility decline by impairing reproductive neuroendocrine hormone release through inducing apoptosis and inhibiting the ERK signal in the hypothalamic–pituitary region. This study can also provide reference for the damage of copper pollution to the male reproductive system.

## Introduction

Wilson’s disease (WD), also known as hepatolenticular degeneration (HLD), is an autosomal recessive inherited disease of a copper metabolism disorder caused by ATP7B gene mutation (1). According to statistics, the incidence rate of the world population is approximately 1/100,000-3/100,000 (2). Due to the extensive deposition of copper in the body, the clinical manifestations are mainly extrapyramidal symptoms, abnormal mental behavior, liver and kidney function impairment, and a corneal pigment ring (K-F ring) (3). In addition, the male reproductive system damage caused by it is very common but easy to be ignored. Clinically, copper complexing agents such as sodium dimercaptopropanesulfonic acid are often used to promote copper excretion *in vivo* to improve the corresponding symptoms (4).

At present, little is known about the reproductive system damage of male WD, in clinical practice, and the changes of sex hormone levels are mostly observed in clinical practice. In-depth studies are few, and large-sample investigations are lacking. Studies have found that male WD patients may involve the pituitary gland to damage the function of the hypothalamic–pituitary–testicular (HPT) axis, leading to the abnormal secretion of sex hormones, infertility, breast development, and other manifestations. Among 26 male WD patients, 21 showed signs of damage to the reproductive system (5). Copper deposition can directly affect the central nervous system, mainly involving the bilateral basal ganglia, thalamus and brainstem, and so on, and copper-induced liver damage can also lead to the morphological and physiological changes of the brain structure (6, 7). However, the precise regulatory mechanism of HPT axis dysfunction in male WD patients has not been investigated. We propose that the deposit of copper in the hypothalamus and pituitary gland may lead to the dysfunction of the HPT axis, abnormal reproductive hormone levels, and a series of the symptoms of reproductive function impairment. Therefore, in this study, male TX mice with the ATP7B gene defect background were used to investigate whether there is a dysfunction of the HPT axis and the damage mechanism and to observe the situation after copper drainage intervention with dimeric-succinate. The copper content, morphology, ultrastructure, and apoptosis of the hypothalamus and pituitary were evaluated. By analyzing the gonadotropin-releasing hormone (GnRH), follicle-stimulatin hormone FSH, luteinizing hormone (LH), testosterone (T) content, fertility, apoptosis-related proteins in hypothalamus, and the pituitary tissues and expression levels of extracellular signal-regulated kinase (ERK)1/2 and P-ERK1/2 proteins, the mechanism of male fertility decline in the TX mouse model of WD was studied, providing an objective theoretical basis for clinical male WD complicated with reproductive system function impairment.

## Materials and methods

### Experimental animals

A total of 60 homozygous male mice and 20 wild-type male mice bred from C3He-ATP7b^tx-j^ mice purchased from Jackson Laboratory in the United States, weighing 19 ± 5 g, were used in the Specific pathogen free (SPF) environment of the Animal Experiment Center, School of Life Sciences, University of Science and Technology of China. After feeding to 12 weeks of age, homozygous male mice were randomly divided into the WD group, DMSA group, and PMSG group, with 20 mice in each group. Wild-type male mice comprise the NC group. The experiment was reviewed by the animal Ethics Committee of Anhui University of Chinese Medicine.

### Drug administration and sampling

Three tablets of dimercaptosuccinic acid capsules were dissolved in 100 ml of warm water, and the DMSA group was given the drug dose of 20 ml of liquid/kg body weight. The NC group, WD group, and PMSG group were given the same amount of normal saline once every morning for 14 days. The PMSG group was intraperitoneally injected with 5IU PMSG (Solarbio, Beijing, China) every morning for 14 consecutive days.

On the first day after the end of the intragastric cycle, the abdominal aorta was anesthetized by the intraperitoneal injection of 1% pentobarbital sodium (50 mg/kg), and blood was collected for the ELISA test. The skull was removed from the middle to both sides before cutting the top of the skull along the median line to the coronal suture. The optic nerves on both sides were cut off with ophthalmic scissors and explored to the base of the skull. The whole brain tissue was slowly removed and placed on an ice bag with the abdomen facing upwards. It was quickly frozen stored at -80°C for tissue copper determination and Western blotting, 5% glutaraldehyde fixative (precooled at 4°C) for electron microscopy and 4% paraformaldehyde (PFA) fixation, and prepared paraffin sections for HE staining and TdT-mediated dUTP nick end labeling (TUNEL) staining.

### Determination of copper content in tissues

The hypothalamus and pituitary tissues were respectively placed in 250 ml of Kellogg flask, and 4ml of HNO_3_ and 1 ml of HCIO_4_ was added to soak overnight. Then, 3 ml of an hNO_3_-HciO_4_ mixed acid digestion solution prepared at 4:1 was added to digest on the electric heating plate until colorless and transparent, and a small amount of deionized water was added to drain acid. After cooling, deionized water was used for constant volume to 10 ml. The same amount of mixed acid digestion solution was taken as the digestion sample, and the blank sample was prepared in the same way. The standard application solution was prepared with a copper (Cu) standard reserve solution. The standard series of copper elements, a reagent blank solution, and a digested sample solution were imported into an atomic absorption spectrophotometer for repeated determination, and the data were exported and analyzed.

### Hematoxylin and eosin staining

The hypothalamus and pituitary tissues were fixed in a 4% PEA solution for 24–48 h, dehydrated and transparent, soaked in paraffin, then embedded, successively sliced 4-µm thick and glued to the glass slides, dewaxed, immersed in hematoxylin (B006, Ebiogo, Anhui, China) for 2–5 min, then cyanated, then placed in an eosin dye (B005, Ebiogo, Anhui, China) medium for 2 min, dehydrated and transparent, and then sealed. The histopathological structure of the hypothalamus and pituitary gland was observed under a microscope (CX41; OLYMPUS, Tokyo, Japan).

### Transmission electron microscope

Hypothalamus and pituitary tissues were cut into 1 mm^3^ and fixed in a 2.5% glutaraldehyde solution for 6–12 h. After washing in phosphate-buffered saline (PBS, pH 7.4), they were fixed in 1% osmium (18456, TED PELLA INC, California, USA) after 1–2-h gradient ethanol dehydration, and then respectively in epoxy propane (M25514, Myrell, Shanghai, China), 1:1 prepared epoxy propane and epoxy resin (18042, TED PELLA INC, California, USA) were soaked for 30 min and 1.5 h, then embedded in epoxy resin, and baked at 40°C and 60°C ovens for 12 and 48 h, respectively. Continuous slices with a thickness of 70 nm were dyed and washed with double-steamed water. Transmission electron microscopy (JEM1400, JEOL, Tokyo, Japan) was used for observation and film taking (Morada G3, EMSIS, Munster, Germany). The structure and morphology of mitochondria rough endoplasmic reticulum Golgi apparatus in neurons and gonadotropin cells were observed. The abnormal mitochondria rate is the number of abnormal mitochondria/total number of mitochondria (8).

### TdT-mediated dUTP nick end labeling staining

Sections were dewaxed and hydrated; then, 20-µg/ml protease K (B030, Ebiogo, Anhui, China) was dropped and incubated in a 37°C incubator (GNP-9080, Sanfa, Shanghai, China), and then washed with PBS for three times. The TUNEL detection solution was prepared by mixing the TdT enzyme and a fluorescent labeling solution at a ratio of 1:9 and dropped onto the slices. After incubation at 37°C for 1 h, the samples were washed with PBS for three times. The tablets were then sealed with anti-fluorescence quenching tablets (including 4',6-diamidino-2-phenylindole DAPI) (B024, Ebiogo, Anhui, China) and observed and photographed under a fluorescence microscope. Five sections were selected from each group, and three fields were selected from each section. ImageJ software was used to calculate the number of positive (green) apoptotic cells and total cells (blue) in the images, and the ratio of apoptotic cells to total cells ×100% was recorded as the apoptosis rate.

### Western blot analysis

Approximately 10-mg hypothalamus and pituitary tissue samples were lysed with RIPA buffer containing PMSF (P0013B, Beyotime, Shanghai, China) and homogenized by ice bath. Total protein was extracted and protein concentration in the samples was determined. Approximately 6% or 12% sodium dodecyl sulfate-polyacrylamide gel electrophoresis was added to the samples, 5–10 μl were added to each well. Electrophoresis was applied at a constant pressure of 80 v for 1 h; then, the model was transferred to a polyvinylidene fluoride membrane (IPVH00010, Millipore, Massachusetts, USA), A Western blocking solution containing 5% skim milk powder was added and shocked at room temperature for 2 h; then, ERK1/2 (ab201015, anti-rabbit, 1∶1,000; Abcam, Cambridge, UK), P-ERK1/2 (SC-136521, resistant rabbit, 1∶1,000; Santa Cruz, CA, USA), Bax (ab32503, resistant rabbit, 1:5,000; Abcam, Cambridge, UK), Caspase-3 (ab184787, anti-rabbit, 1:2000; Abcam, Cambridge, UK) and Bcl-2 (ab32124, rabbit resistant, 1∶1,000; Abcam, Cambridge, UK) antibodies, sealed at 4°C overnight. The membrane was then incubated with a secondary antibody (goat anti-igG, ZB-2301, Zsbio, Shanghai, China) coupled with horseradish peroxidase (HRP) for 1.2 h at room temperature. The ECL luminescence kit (340958, Thermo, Massachusetts, USA) was used for protein detection. The X-ray film was developed by scanning the X-ray film. Protein levels were then analyzed using ImageJ software.

### Enzyme-linked immunosorbent assay

Blood was taken from the abdominal aorta, placed at room temperature and centrifuged at 3, 000 rpm for 20 min. GnRH(JYM0505Mo, Genmei, Wuhan, China), LH(JYM0341Mo, Genmei, Wuhan, China), and T(JYM0373Mo, Genmei,Wuhan, China) were analyzed using ELISA kits according to the manufacturer’s instructions. The absorbance (OD) was measured at 450 nm. The hormone levels in the samples were calculated according to the standard data curve.

### Fertility test

Male mice in the NC group, WD group, DMSA group, and PMSG group were caged with normal female mice of the same generation at a ratio of 1:2, respectively, and observed two-to-three estrus cycles. The formation of a vaginal plug in female mice was observed every morning. If there was any, female mice were removed and placed in a cage separately. The pregnancy rate and litter size of female mice were recorded (pregnancy rate = the number of pregnant mice/number of female mice with a vaginal plug).

### Statistical analysis

SPSS 23.0 software was used for statistical analysis. The measurement data were expressed as mean ± standard deviation $\left(\bar{x}\pm s\right)$ . One-way ANOVA was used for comparison between multiple groups, and the LSD test was used for post-comparison.

## Results

### Copper content in hypothalamus and pituitary tissues

The content of copper in hypothalamus and pituitary tissues was measured. The results showed that the content of copper in hypothalamus and pituitary tissues in WD group was significantly higher than that in NC group (*P* < 0.05). Compared with WD group, the content of copper in hypothalamus and pituitary tissues of male mice in DMSA group was significantly decreased (*P* < 0.05)(**Table 1**).

**Table 1:** Comparison of copper content in the hypothalamus and pituitary tissues of male mice in each group ( $\bar{x}\pm s$ , n=5).

Group	>Hypothalamus (µg/g)	>Pituitary (µg/g)
NC	3.95 ± 0.41	3.26 ± 0.28
WD	6.47 ± 0.19^*^	6.02 ± 0.37^*^
DMSA	4.70 ± 0.34^#^	4.53 ± 0.28^#^

Compared with the NC group, *P < 0.05; Compared with the WD group, ^#^P < 0.05

### Histopathology in hypothalamus and pituitary

**Figure 1:**
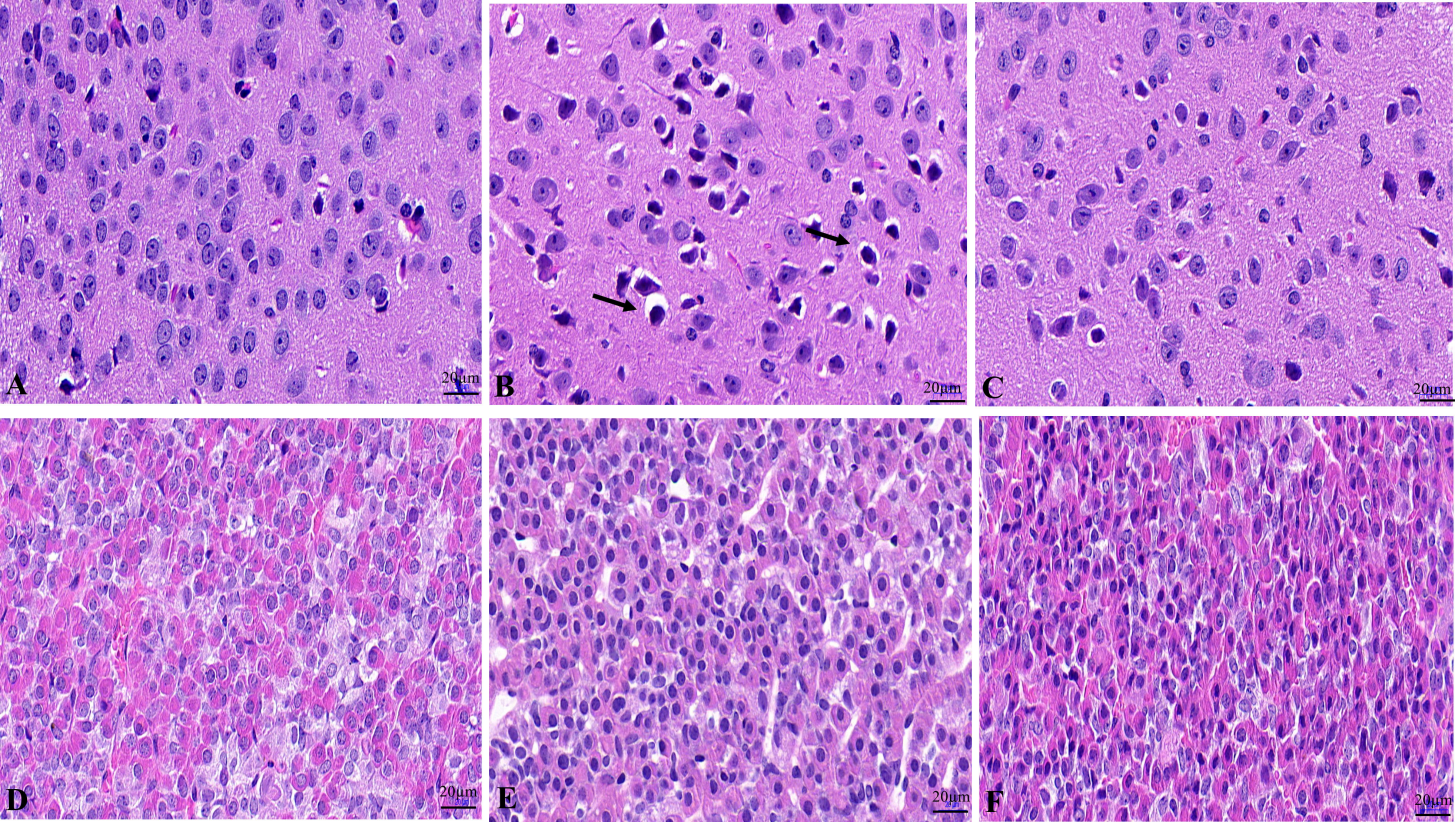
Histopathological changes of the hypothalamus and pituitary in male mice. Histopathological changes of hypothalamus in NC group **(A)**, WD group **(B)** and DMSA group **(C)**. Histopathological changes of pituitary in NC group **(D)**, WD group **(E)** and DMSA group **(F)**.

The results showed that the hypothalamic nerve cells in the NC group were normal in size and shape, with a complete nucleus, clear structure, and uniform opaque contents (**Figure 1A**). The tissue structure of pituitary gland is normal, and the cells are closely and neatly arranged with normal morphology and uniform distribution (**Figure 1D**). Hypothalamic nerve cells in the WD group showed swelling, and the black arrow showed the cytoplasmic swelling of nerve cells accompanied by nuclear pyknosis (**Figure 1B**). Pituitary cells were disordered, with loose tissue structure and edema (**Figure 1E**). The degree of hypothalamic nerve cell edema and nuclear pyrosis in the DMSA group was less than that in the model group (**Figure 1C**). Compared with the model group, the degree of pituitary tissue looseness was reduced, and the cells were arranged neatly (**Figure 1F**).

### Ultrastructure in hypothalamic neurons and pituitary gonadotropin cells

**Figure 2:**
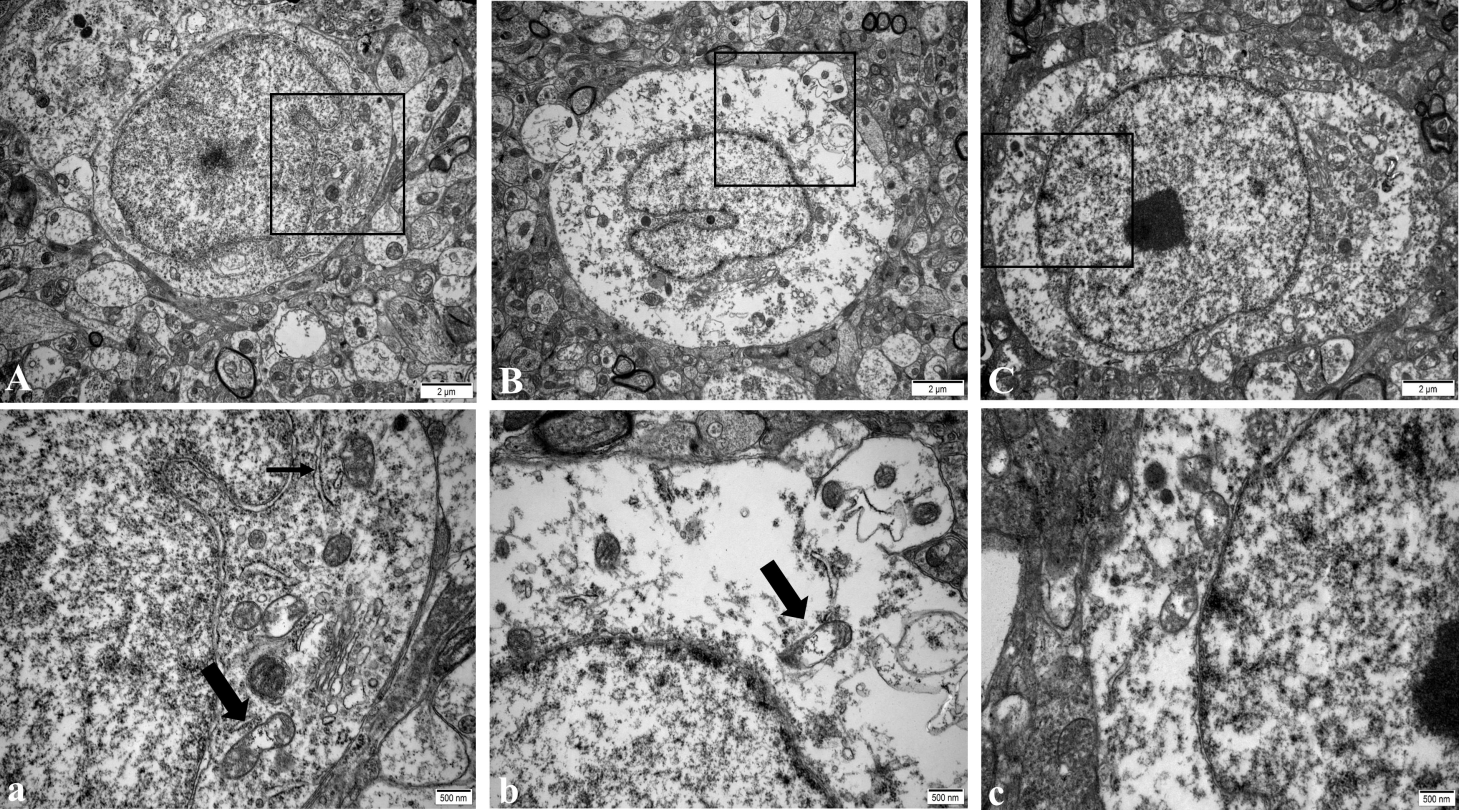
Ultra structure of hypothalamic neurons in male mice of NC group **(A)**, WD group **(B)** and DMSA GROUP **(C)**. The black rectangle corresponds to the local magnification of each group of neurons **(a–c)**. In panel **(a),** the coarse arrow indicates mitochondria, and the thick arrow indicates rough endoplasmic reticulum. The arrow in panel **(b)** shows mitochondria with missing cristae.

In the NC group, the regular morphology of hypothalamus neurons, uniform chromatin distribution, many uniformly distributed free ribosomes in the cytoplasm, the clear structure of most mitochondria, and normal Golgi apparatus and rough endoplasmic reticulum were observed (**Figure 2A**). In the WD group, a large number of ribosome particles were lost in neurons, organelles were significantly reduced, structural destruction disappeared, and mitochondrial swelling appeared with vacuolation and crest disappearance (**Figure 2B**). Compared with the model group, the number of ribosome particles in neurons in the DMSA group was increased, and the mitochondrial structure was recovered. Abnormal degeneration and crest fracture were still found in some neurons, and the Golgi apparatus could be seen (**Figure 2C**).

**Figure 3:**
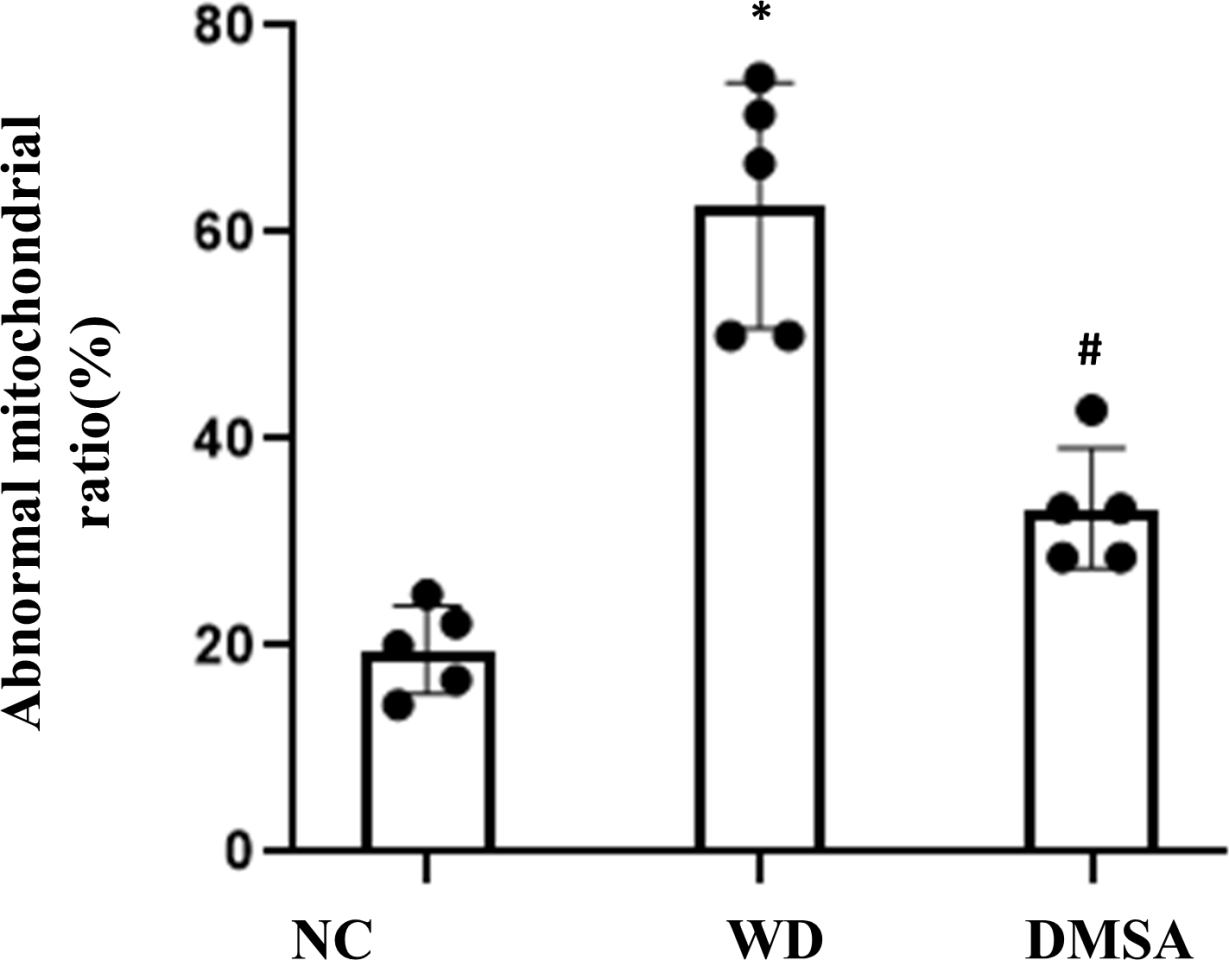
Ratio of abnormal mitochondria in hypothalamic neurons. Data are presented as mean ± SD.(*P<0.05, NC group vs. WD group; ^#^P<0.05, WD group vs. DMSA group).

Compared with the NC group, the number of normal mitochondria in hypothalamus neurons in the WD group was significantly decreased (*P*<0.05), and the number of abnormal mitochondria was significantly increased (*P*<0.05). Compared with the WD group, the number of normal mitochondria in the DMSA group was significantly increased (*P* < 0.05), and the number of abnormal mitochondria was significantly decreased (*P* < 0.05) (**Figure 3**).

**Figure 4:**
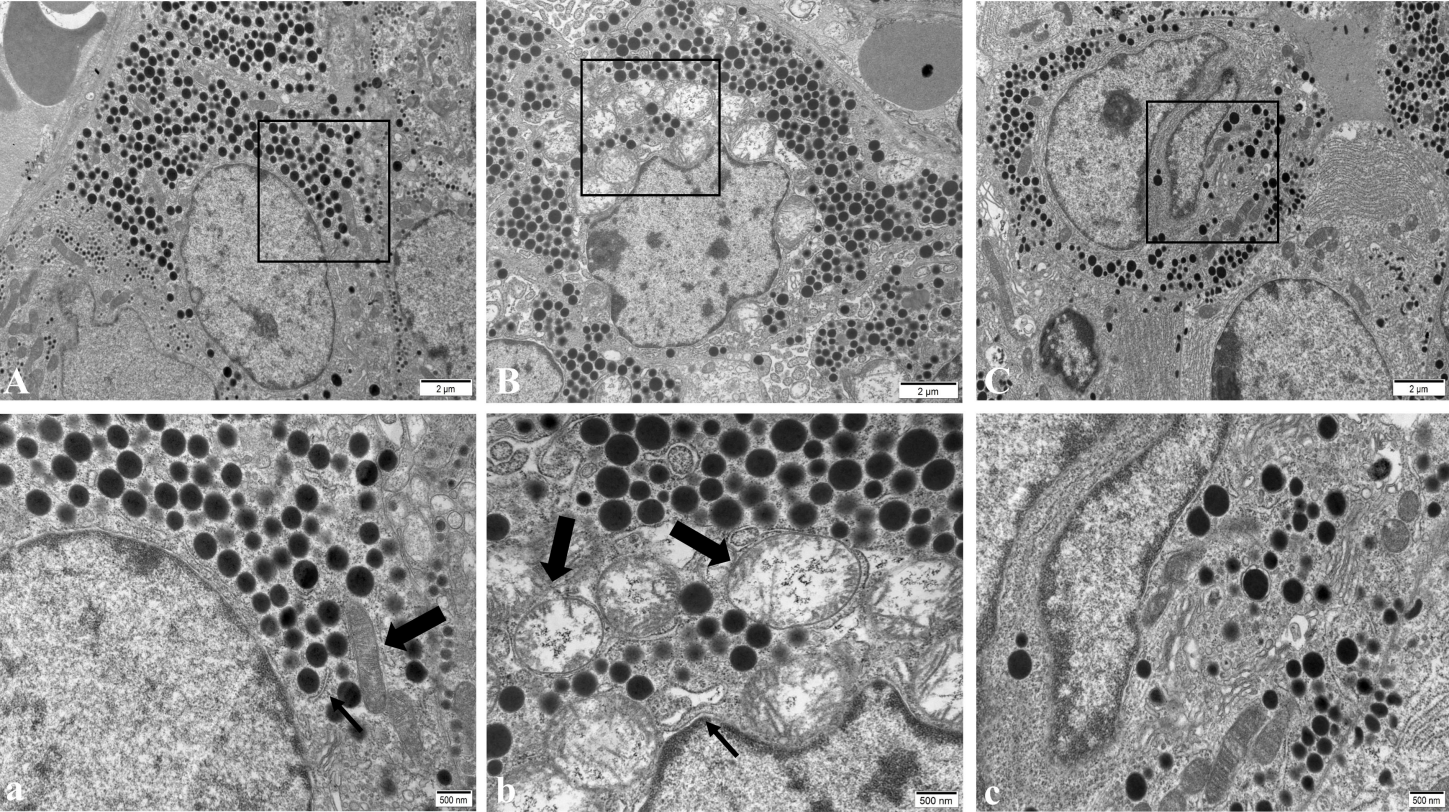
Ultrastructure of pituitary gonadotropin cells in male mice of NC group **(A)**, WD group **(B)** and DMSA group **(C)**. The black rectangle corresponds to the local magnification of each group of gonadotropin cells **(a–c)**. In panel **(a)**, the coarse arrow shows mitochondria and the thick arrow shows the rough endoplasmic reticulum. In panel **(b)**, the coarse arrow indicates the swollen and degenerated mitochondria and the thick arrow indicates the enlarged rough endoplasmic reticulum.

In the NC group, pituitary gonadotropin cells had regular morphology, a smooth nuclear membrane, normal chromatin distribution, a regular distribution of rough endoplasmic reticulum, more ribosome particles attached on the surface, a clear mitochondrial structure, and spherical secreting particles (**Figure 4A**). In the WD group, gonadotropin cells showed nuclear pyknosis, a heterochromatin edge set, obvious mitochondrial swelling, internal crest fracture, rough endoplasmic reticulum fracture and expansion, and apoptotic tendency (**Figure 4B**). After DMSA intervention, the cell morphology was improved compared with the model group, and the number of normal mitochondria was increased compared with the model group. A small amount of mitochondrial vacuolar degeneration and crest loss were still observed, and rough endoplasmic reticulum was slightly expanded (**Figure 4C**).

**Figure 5:**
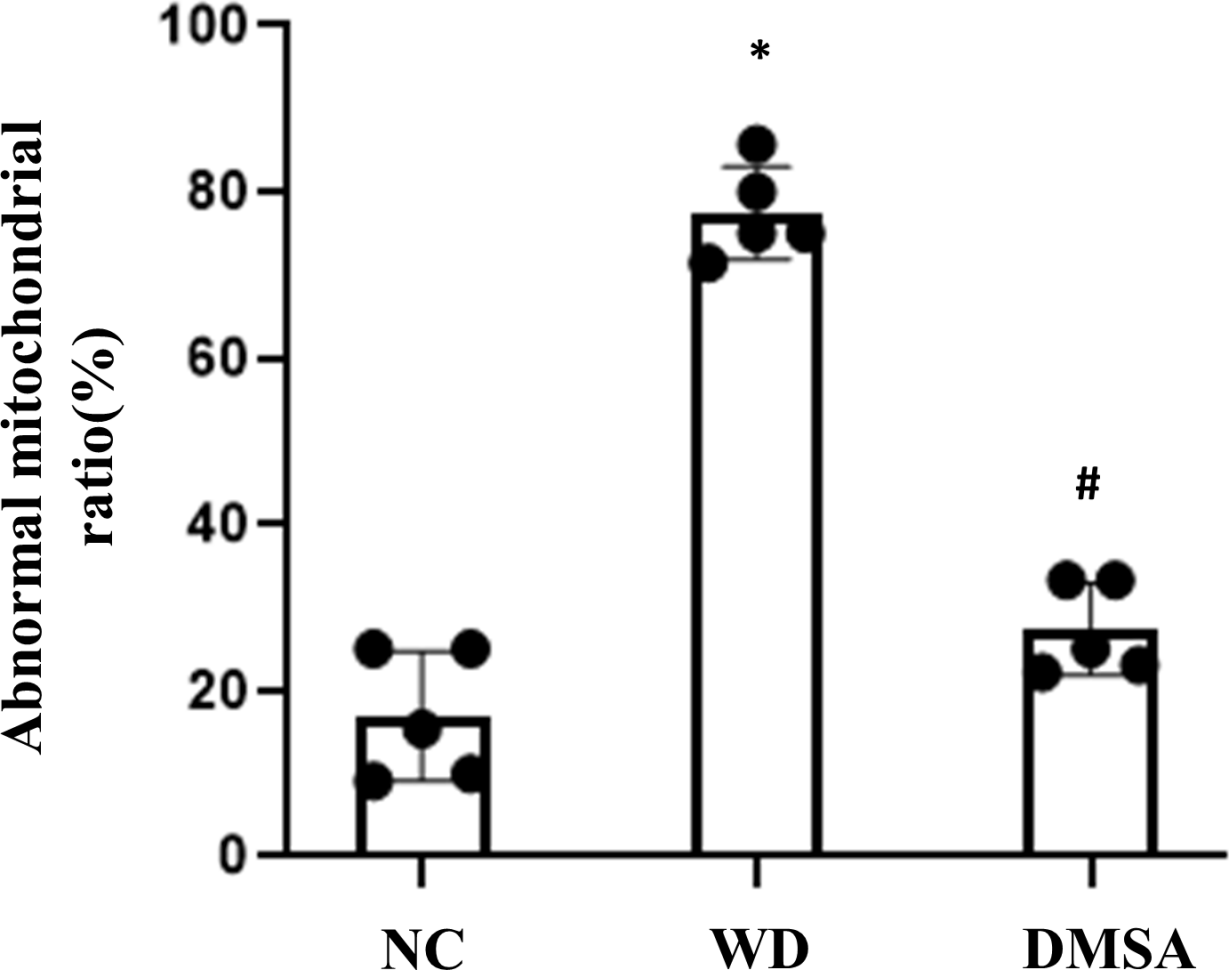
Ratio of abnormal mitochondria in pituitary gonadotropin cells. Data are presented as mean ± SD.(*P<0.05, NC group vs. WD group; ^#^P<0.05, WD group vs. DMSA group).

Compared with the NC group, the number of the normal mitochondria of pituitary gonadotropin cells in the WD group was significantly decreased (*P* < 0.05), and the number of abnormal mitochondria was significantly increased (*P* < 0.05). Compared with the WD group, the number of normal mitochondria in the DMSA group was significantly increased (*P* < 0.05), and the number of abnormal mitochondria was significantly decreased (*P* < 0.05) (**Figure 5**).

### Apoptosis in hypothalamic and pituitary

**Figure 6:**
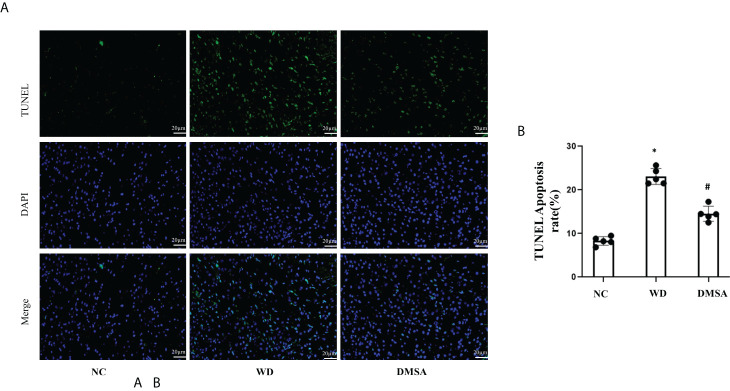
Apoptosis of hypothalamus neurons in male mice ( $\overset{¯}{\text{x}}\pm$ ±*s*). Apoptosis analysis of hypothalamus neurons in male mice **(A, B)**. Data are presented as mean ± SD.(*P<0.05, NC group vs. WD group; ^#^P<0.05, WD group vs. DMSA group).

Hypothalamic TUNEL staining results showed that the number of apoptotic neurons in the hypothalamic tissue of WD male mice was significantly increased compared with the NC group (*P*< 0.05). The apoptosis of hypothalamus neurons in the DMSA group was significantly decreased compared with that in the WD group (*P* < 0.05) (**Figures 6A, B**).

**Figure 7:**
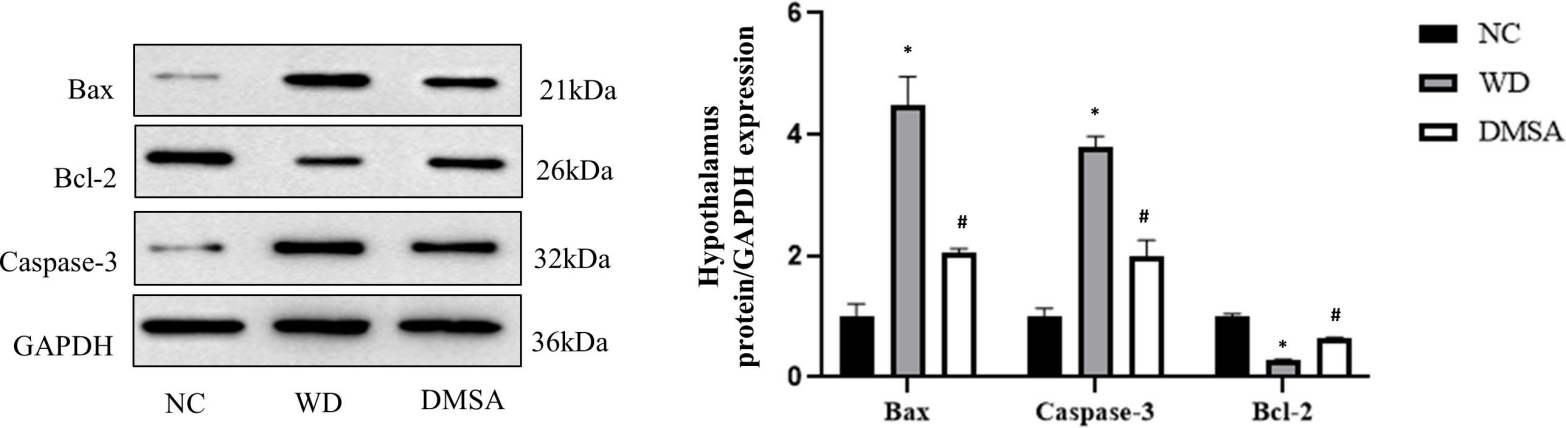
Expression levels of Bax, Caspase-3 and Bcl-2 proteins in the male hypothalamus. Data are presented as mean ± SD.(*P<0.05, NC group vs. WD group; ^#^P<0.05, WD group vs. DMSA group).

The Western blotting results of hypothalamus showed that the expression levels of pro-apoptotic proteins Bax and Caspase-3 were significantly increased in the hypothalamus of male mice in the WD group, while the expression levels of anti-apoptotic protein Bcl-2 were significantly decreased (*P*< 0.05). Compared with the WD group, the expression levels of pro-apoptotic proteins Bax and Caspase-3 in the DMSA group were significantly decreased, while the expression level of the anti-apoptotic protein Bcl-2 was significantly increased (*P* < 0.05) (**Figure 7**).

**Figure 8:**
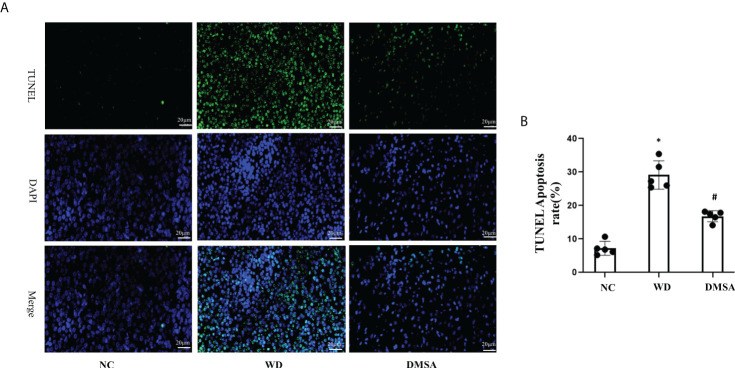
Apoptosis analysis of pituitary cells in male mice **(A, B)**. Data are presented as mean ± SD.(*P<0.05, NC group vs. WD group; ^#^P<0.05, WD group vs. DMSA group).

The results of pituitary TUNEL staining showed that compared with the NC group, the number of apoptotic neurons in the pituitary tissue of WD male mice was significantly increased (*P*< 0.05). Compared with the WD group, the apoptosis of pituitary cells in the DMSA group was significantly decreased (*P* < 0.05) (**Figures 8A, B** **)**

**Figure 9:**
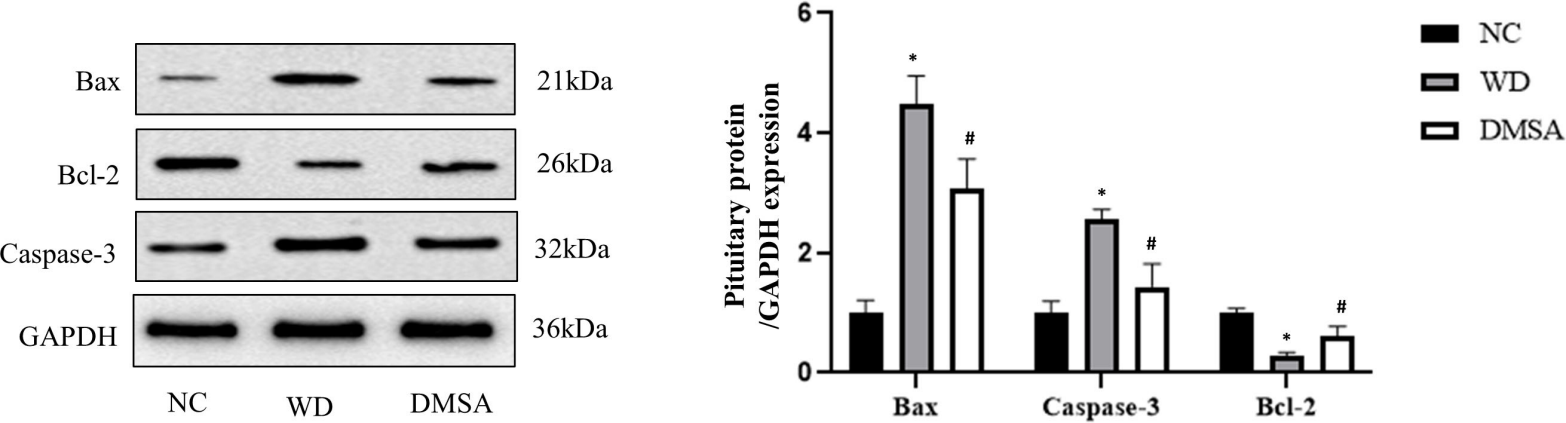
Expression levels of Bax, Caspase-3 and Bcl-2 proteins in the male pituitary. Data are presented as mean ± SD. (*P<0.05, NC group vs. WD group; ^#^P<0.05, WD group vs. DMSA group).

Pituitary Western blotting results showed that the expression levels of pro-apoptotic proteins Bax and Caspase-3 in the pituitary tissues of male mice in the WD group were significantly increased, while the expression levels of the anti-apoptotic protein Bcl-2 were significantly decreased (*P*< 0.05). Compared with the WD group, the expression levels of pro-apoptotic proteins Bax and Caspase-3 in the DMSA group were significantly decreased, while the expression level of the anti-apoptotic protein Bcl-2 was significantly increased (*P* < 0.05) (**Figure 9**).

### Extracellular regulatory protein kinase 1/2 phosphorylation in hypothalamus and pituitary

**Figure 10:**
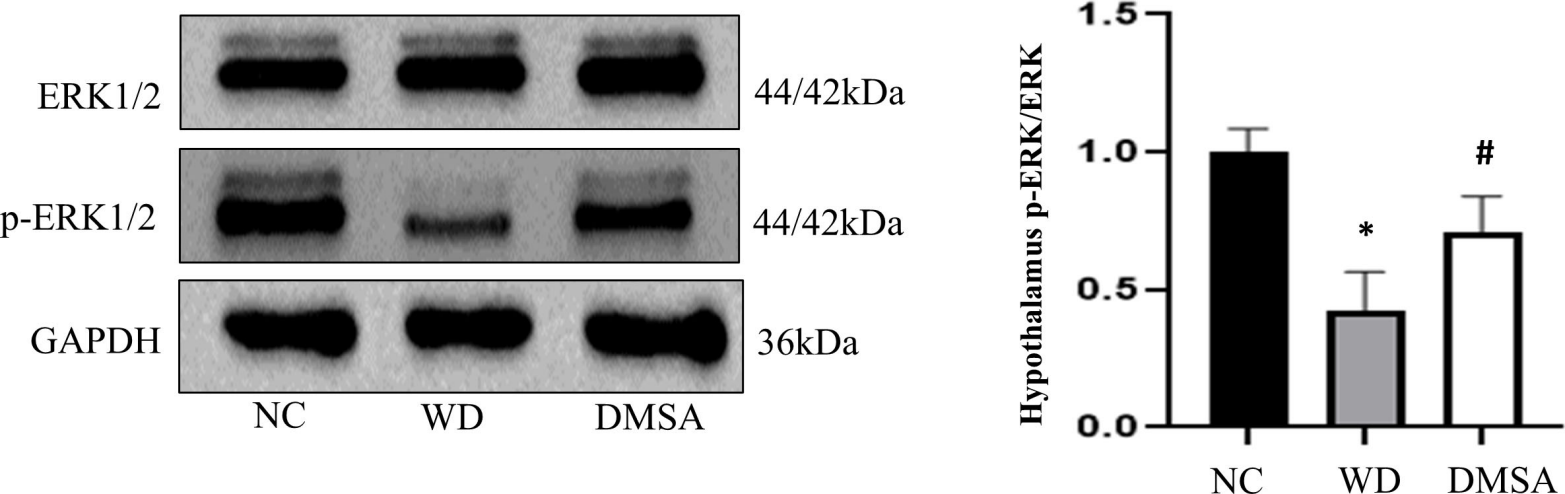
Extracellular regulatory protein kinase (ERK) 1/2 phosphorylation in the male hypothalamus. Data are presented as mean ± SD.(*P<0.05, NC group vs. WD group; ^#^P<0.05, WD group vs. DMSA group).

We found that the expression level of P-ERK1/2 in the hypothalamus of male mice in the WD group was significantly downregulated compared with that in the NC group (*P* < 0.05). The expression level of P-ERK1/2 was significantly increased after DMSA intervention (*P* < 0.05). There was no significant difference in the expression level of ERK1/2 in the hypothalamus of male mice (**Figure 10**)

**Figure 11:**
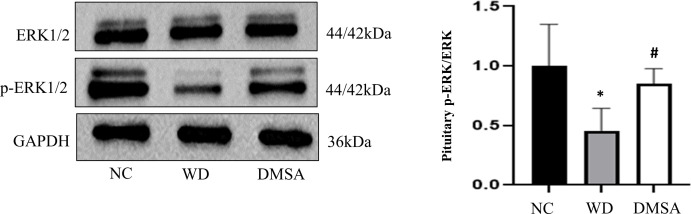
ERK1/2 phosphorylation in the male pituitary. Data are presented as mean ± SD.(*P<0.05, NC group vs. WD group; ^#^P<0.05, WD group vs. DMSA group).

Western blotting results showed that compared with NC, the expression level of P-ERK1/2 in the pituitary tissues of male mice in the WD group was significantly downregulated (*P* < 0.05). The expression level of P-ERK1/2 was significantly increased after DMSA intervention (*P* < 0.05).There was no significant difference in the expression of ERK1/2 in pituitary tissues among all groups (**Figure 11**).

### The serum gonadotropin-releasing hormone, follicle-stimulating hormone, luteinizing hormone, and testosterone levels

The contents of GnRH, FSH, LH, and T in the serum of each group were detected. The results showed that compared with the NC group, serum GnRH, FSH, LH, and T levels in the WD group were significantly decreased (*P* < 0.05). Serum GnRH, FSH, LH, and T levels in the DMSA group were significantly higher than those in the WD group (*P* < 0.05) (**Table 2**).

**Table 2:** Comparison of serum GnRH, FSH, LH and T levels in three group ( $\bar{x}\pm s$ , n=5).

Group	GnRH(pg/ml)	FSH (ng/ml)	LH (ng/ml)	T (ng/ml)
NC	298.58 ± 12.73	51.25 ± 3.54	8.16 ± 0.41	3.01 ± 0.13
WD	184.54 ± 26.97*	29.30 ± 3.50*	5.42 ± 0.52*	1.49 ± 0.09*
DMSA	243.23 ± 3.36^#^	45.28 ± 3.49^#^	7.08 ± 0.32^#^	2.31 ± 0.15^#^

Compared with the NC group, *P < 0.05; Compared with the WD group, ^#^P < 0.05

### Fertility test

The fertility of male mice in each group was compared. The results showed that compared with the NC group, the pregnancy rate and litter size of the WD group were significantly decreased (*P*< 0.05). The pregnancy rate and litter size of female mice mated with the DMSA group and PMSG group were significantly increased (*P* < 0.05). Compared with the PMSG group, there were no significant differences in the pregnancy rate and litter size between the DMSA group and PMSG group (*P* > 0.05) (**Table 3**).

**Table 3:** Comparison of fertility of male mice in each group ( $\bar{x}\pm s$ , n=10).

Group	Fertility rate (%)	Pups per litter
NC	80	10.38 ± 2.45
WD	31.59	4.83 ± 1.6*
DMSA	58.82	8.10 ± 2.47^#^
PMSG	66.67	8.58 ± 1.88^#^

Compared with the NC group, *P < 0.05; Compared with the WD group, ^#^P < 0.05

## Discussion

In addition to extrapyramidal symptoms and damage to liver functions, male WD patients can also show the symptoms of reproductive system damage such as infertility, feminine development, and sexual dysfunction (9, 10). The functions of the male reproductive system are mostly regulated by the HPT axis (11). This axis mainly regulates the synthesis and secretion of GnRH, LH, FSH, and gonadal steroid hormones. The HPT axis can maintain the dynamic balance of serum reproductive hormone levels through a closed-loop feedback mechanism and maintain the relative stability of the reproductive endocrine system (12). The current clinical treatment is mainly used to remove toxic copper stored in tissues, and copper complexing agents that promote copper excretion are mostly used 13, 14). Sodium dimercaptopropane sulfonate is an oral metal chelating agent that can be excreted in complexes with a variety of heavy metals and is commonly used in the treatment of heavy metal poisoning, including those caused by lead, mercury, cadmium, and copper (15, 16). Studies have shown that treatment with sodium dimercaptopropane sulfonate for copper drainage can reduce metal deposition in the brain, thus improving the corresponding neurological symptoms (17).

**Figure 12:**
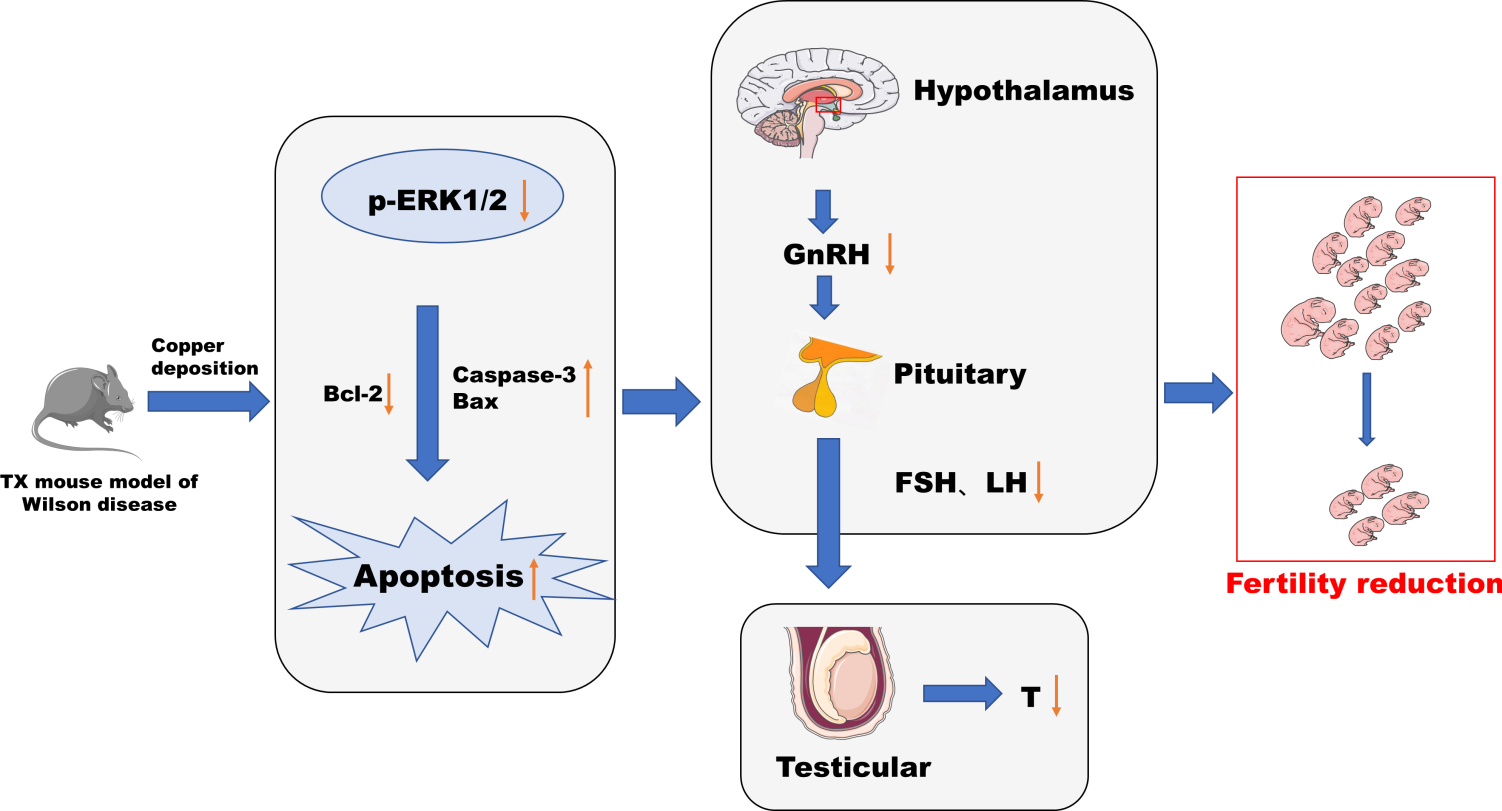
Conclusion diagram.

In this study, male TX mice with an ATP7B gene defect background were used as WD model mice (18, 19), and it was found that the content of copper in hypothalamus and pituitary tissues, abnormal tissue morphology and organelle ultrastructure, increased apoptosis rate, decreased serum GnRH, LH, FSH, T, and fertility decreased significantly, and the phosphorylation level of ERK1/2 was significantly downregulated in TX mice with WD. After copper discharge by DMSA, the copper content in hypothalamus and pituitary tissues decreased, the tissue morphology and organelle ultrastructure improved, the cell apoptosis rate decreased, and the serum GnRH, LH, FSH, and T levels increased. The significantly increased phosphorylation levels of ERK1/2 in the hypothalamus and pituitary tissues suggested that the HPT axis function was impaired in male TX mice with WD. The mechanism might be that copper deposition inhibited the phosphorylation of the ERK1/2 signaling pathway and promoted the expression of downstream proapoptotic proteins The mechanism might be that copper deposition inhibits phosphorylation of the ERK1/2 signaling pathway and promotes the expression of the downstream pro-apoptotic proteins Bax and Caspase-3 and inhibits the expression of the anti-apoptotic protein Bcl-2 through the ERK signaling pathway. This induces apoptosis of hypothalamic and pituitary cells, which affects the release of reproductive neuroendocrine hormones and leads to reduced fertility in male mice (**Figure 12**).

### Effect of Wilson’s disease or copper deposition on male reproduction

Previous studies have found that male WD patients may suffer from reproductive system damage such as infertility, abnormal breast development, and reproductive endocrine (5). In addition, studies have found that the sperm motility of male WD patients is significantly lower than that of normal men, among which, those with poor disease control may have severe oligoasthenoteratozoospermia (9) but there are still no findings yet regarding the study of fertility changing WD. This study by both the male and female rat-pairing fertility of male mice and the cage experiment observation, discovery, and WD group of female mating its conception rate and litter size were significantly decreased, while the DMSA copper after the intervention, and its mating female conception rate and litter size being significantly elevated, shows that the WD group decreased fertility in both males and WD may impair male fertility to some extent. The fertility of male mice injected with PMSG was also significantly improved compared with WD group. Pregnant Mare Serum Gonadotropin (PMSG) has dual activities similar to FSH and LH, and studies have shown that PMSG can play the function of a pituitary gonadotropin and promote the development of fine tubules and the differentiation of sex cells (20). These results indicate that the decreased fertility of the WD group may be related to the abnormal secretion of the hypothalamic–pituitary reproductive hormone. A large number of basic studies (21) have confirmed that excessive copper can cause certain damage to the male reproductive system, but there are few studies on the secretion of reproductive hormones. Studies have shown that a low-dose copper exposure of 60 μg can significantly reduce serum FSH and LH levels in mice (22). Some studies have also found that chronic copper exposure can lead to sperm dysplasia in mice but has no effect on the T level (23). In this study, it was found that the serum levels of GnRH, FSH, LH, and T in the WD group were significantly lower than those in the normal control group, and the serum levels of GnRH, FSH, LH, and T were significantly increased after copper drainage intervention by DMSA. The decrease of T level in the model group may be caused by pituitary function impairment and a body feedback regulation mechanism, leading to decreased T secretion. Among them, FSH and LH are regulated by GnRH secretion (24), and both can promote sperm maturation and androgen secretion, while T, as an important androgen in a male body, plays an important role in maintaining the male sexual function and desire. WD can affect the function of the male reproductive system and reduce fertility.

### Effect of Wilson’s disease or copper deposition on histopathology and ultrastructure

Many previous studies have found that copper deposits in the liver can lead to pathological changes such as hepatocellular balloon degeneration, steatosis and anucleosis (25) and can also accumulate in the brain tissue, resulting in brain atrophy and other structural and functional changes (26). Only a few studies have found that WD may involve the hypothalamus or pituitary gland, resulting in impaired HPT axis function and reproductive function (5). However, there are no studies on the copper content, histomorphology, and ultrastructure of hypothalamus and pituitary tissue in WD. In this study, it was found that the copper content in hypothalamus and pituitary tissues of male mice in the WD group was significantly higher than that in normal control group, indicating that there was obvious copper deposition in the hypothalamus and pituitary tissues of male TX mice with WD. The histopathological structure and organelle ultrastructure were observed. It was found that the hypothalamic nerve cells were swollen and had nuclear pyknosis, and the pituitary tissue structure was loose and cells were arranged disorderly in the male mice of the WD model group. Hypothalamic neurons appear to have a large number of ribosome particle loss, mitochondria swelling disappeared in vacuoles, crest, pituitary gonadotropins cells appear obvious nucleus pycnosis, heterochromatin edge set, mitochondria crest fracture, fracture, rough endoplasmic reticulum expansion, shows that the organizational structure destruction signs, for male Wilson disease pathology basis is provided for HPT axis dysfunction. After DMSA copper drainage intervention, the copper content in the hypothalamus and pituitary tissues of male TX mice with WD was significantly decreased, the degree of tissue morphological damage was significantly alleviated, and the organelle structure was also improved. It was further confirmed that excessive copper deposition in the hypothalamus and pituitary gland may damage histomorphology and the organelle ultrastructure to a certain extent. The reason may be that excessive copper ions can produce a large amount of H_2_O_2_ through Fenton’s reaction, which reduces the activity of antioxidant enzymes, resulting in damage to the structure and function of cell membranes and the abnormal expression of mitochondria-related proteins in the brain, resulting in the disruption of mitochondrial division and fusion, leading to dysfunction (27). Hypothalamic neurons mainly regulate GnRH secretion. If the mitochondrial structure of their nerve cells is damaged and energy metabolism is disturbed, cell dysfunction will result. Gonadotropin cells mainly synthesize FSH and LH through the mitochondrial energy supply and rough endoplasmic reticulum (28). The results showed that the ultrastructure of neurons and gonadotropin cells could also affect the synthesis and release of gonadotropin, which was consistent with the results of reproductive hormone levels in this study, and the two had a good correlation.

### Cellular death path induced by Wilson’s disease or copper deposition

Bcl-2 family proteins, including anti-apoptotic proteins and pro-apoptotic proteins, play a key role in the process of cell apoptosis. The Bax protein is present in the cytoplasm of normal cells. When stimulated by a series of apoptotic signals, it initiates the caspase cascade and activates the downstream factor Caspase-3, leading to apoptosis (29, 30). At present, many studies have shown that excessive copper can induce testicular cell apoptosis through mediating oxidative stress and affect reproductive system functions (31). However, the apoptosis of the hypothalamus and pituitary tissue in TX mice with WD has not been observed. The results of this study showed that the apoptosis rate of hypothalamic neurons and pituitary tissue cells in the WD group was significantly increased compared with the normal control group, and the expression levels of pro-apoptotic proteins Bax and Caspase-3 were significantly increased compared with the NC group, while the expression level of the anti-apoptotic protein Bcl-2 was significantly decreased. Compared with the WD group, the apoptosis rate of the DMSA group was significantly decreased, and the expression levels of pro-apoptotic proteins Bax and Caspase-3 were significantly decreased, while the expression level of the anti-apoptotic protein Bcl-2 was significantly increased. These results indicate that excessive copper accumulation in the hypothalamus and pituitary tissues can accelerate the apoptosis of hypothalamus neurons and pituitary cells, which is consistent with the findings by Wang Dongxu et al. that chronic copper poisoning can lead to oxidative stress and mitochondrial dynamics disorder in chicken hypothalamus cells, leading to cell apoptosis (32).

However, the mechanism of cell death caused by excessive copper remains unclear. It is generally believed that copper deposition can induce apoptosis through endoplasmic reticulum stress, mitochondrial autophagy, and oxidative stress (33). It has been found that copper ions can cause changes in mitochondrial membrane permeability, DNA damage, and organelle swelling in the primary hepatocytes of trout, leading to cell necrotic apoptosis (34). The inhibition of NLRP3 inflammosome activation was found to prevent copper overload–induced neuropathological damage in WD mice and to have a protective effect on neurons, suggesting that copper deposition can also induce pyroapoptosis-mediated neurotoxicity in NLRP3-dependent cells (35). In mouse hippocampal neuron models, Maher found that copper induced Glutathione (GSH) depletion and enhanced iron death and decreased cell activity induced by Erastin (ERA), sulfasalazine, and sulfoximine (36). Recent studies have shown that copper directly binds to lipoacylated components through the tricarboxylic acid cycle, resulting in lipoacylated protein aggregation and loss of iron–sulfur cluster proteins, resulting in proteotoxic stress and ultimately cell death, known as copper death (37). Although *in vitro* studies have shown that copper stress mainly causes copper death rather than apoptosis and iron death, more *in vivo* studies, including our study, have shown that apoptosis and other death modes exist simultaneously *in vivo*.

### Effect of Wilson’s disease or copper deposition on ERK1/2 signaling

Extracellular signal-regulated kinase (ERK) 1/2 is one of the most important signaling pathways that promote cell proliferation and differentiation. ERK 1/2 is phosphorylated and activated by MEK1/2(MAP kinase/ERK kinase), and the activated ERK1/2 is transferred from the cytoplasm to the nucleus. ERK phosphorylates some substrates such as transcription factors in the nucleus, which are involved in regulating the proliferation, differentiation, survival, and apoptosis of various cells in brain injury. It is closely related to central nervous system diseases (38, 39). The phosphorylation of ERK 1/2 can activate the cell cycle process, mediate the mitogenic effects of hormones and growth factors, play an important role in regulating the proliferation and differentiation of neurons (40, 41), and inhibit apoptosis (42).

Studies have found that copper can inhibit the phosphorylation of ERK1/2 and promote the apoptosis of cerebellar granule neurons (43). Copper deposition also caused significant reproductive toxicity in mice by disrupting the sex hormone balance and cytotoxicity to human extravillus trophoblast cells through ERK signaling and mitochondrial apoptosis pathways (44). However, no relevant reports have been found in the hypothalamic–pituitary. Studies have shown that the MEK/ERK signaling pathway is involved in the regulation of downstream apoptotic factors (such as Bcl-2, Bax, and Caspase-3) (45–47). Therefore, this study further explored the mechanism of the apoptosis of hypothalamus neurons and pituitary cells and found that the phosphorylation level of ERK1/2 in the hypothalamus and pituitary tissues of male mice in the WD group was significantly downregulated and that in the DMSA group, it was significantly upregulated. These results suggest that copper may accelerate the apoptosis of hypothalamic neurons and pituitary cells by inhibiting the phosphorylation of ERK1/2.

In conclusion, this study found a functional impairment of the HPT axis in WD from the observation of the pathologic level and serum reproductive hormone level. Copper deposition inhibited the phosphorylation of the ERK1/2 signaling pathway in WD, promoted the expression of downstream pro-apoptotic proteins Bax and Caspase-3 through the ERK signaling pathway, inhibited the expression of anti-apoptotic protein Bcl-2, and induced the apoptosis of hypothalamus and pituitary cells. The release of the reproductive neuroendocrine hormone was affected, resulting in decreased fertility of male mice. This study can also provide reference for the study of male reproductive damage caused by environmental copper pollution.

## Conclusion

Our study found that in WD, the function of the HPT axis is impaired, and copper deposition may be related to the inhibition of phosphorylation of ERK 1/2 signaling pathway and the promotion of expression of downstream pro-apoptotic proteins Bax and Caspase-3, as well as the inhibition of expression of anti-apoptotic protein Bcl-2, leading to an increased apoptosis of hypothalamus neurons and pituitary cells. Affect the release of reproductive hormones, resulting in decreased fertility.

## Data availability statement

The original contributions presented in the study are included in the article/supplementary material. Further inquiries can be directed to the corresponding authors.

## Ethics statement

The animal study was reviewed and approved by The Animal Ethics Committee of Anhui University of Traditional Chinese Medicine.

## Author contributions

HH and LW designed the research. TW, QC, KC, FT, JL, and XL performed the research. TW, QC, LW, and HH analyzed the data. TW, LW, and HH wrote the manuscript. All authors contributed to the article and approved the submitted version.

## Funding

This work was supported by the National Natural Science Foundation of China (Grants No. 81971446 and 81673811).

## Conflict of interest

The authors declare that the research was conducted in the absence of any commercial or financial relationships that could be construed as a potential conflict of interest.

## Publisher’s note

All claims expressed in this article are solely those of the authors and do not necessarily represent those of their affiliated organizations, or those of the publisher, the editors and the reviewers. Any product that may be evaluated in this article, or claim that may be made by its manufacturer, is not guaranteed or endorsed by the publisher.
